# Liver Cirrhosis in Chronic Hepatitis B Patients Is Associated with Genetic Variations in DNA Repair Pathway Genes

**DOI:** 10.3390/cancers12113295

**Published:** 2020-11-07

**Authors:** Magda Rybicka, Anna Woziwodzka, Alicja Sznarkowska, Tomasz Romanowski, Piotr Stalke, Marcin Dręczewski, Eloi R. Verrier, Thomas F. Baumert, Krzysztof Piotr Bielawski

**Affiliations:** 1 Department of Molecular Diagnostics, Intercollegiate Faculty of Biotechnology, University of Gdansk and Medical University of Gdansk, Abrahama 58, 80-307 Gdansk, Poland; anna.woziwodzka@ug.edu.pl (A.W.); alicja.sznarkowska@ug.edu.pl (A.S.); t.romanowski@biotech.ug.edu.pl (T.R.); 2 International Centre for Cancer Vaccine Science, University of Gdansk, ul. Wita Stwosza 63, 80-308 Gdansk, Poland; 3 Department of Infectious Diseases, Medical University of Gdansk, ul. Powstania Styczniowego 9b, 81-519 Gdynia, Poland; pstalke@gumed.edu.pl (P.S.); marcin.dreczewski@gumed.edu.pl (M.D.); 4 Inserm, Institut de Recherche sur les Maladies Virales et Hépatiques UMR_S1110, Université de Strasbourg, F-67000 Strasbourg, France; e.verrier@unistra.fr (E.R.V.); thomas.baumert@unistra.fr (T.F.B.); 5 Pôle Hépato-Digestif, Institut Hospitalo-Universitaire, Hôpitaux Universitaires de Strasbourg, 67-000 Strasbourg, France

**Keywords:** HBV, liver cirrhosis, genetic polymorphism, DNA repair, *XRCC1*, *ERCC2*

## Abstract

**Simple Summary:**

As DNA repair enzymes affect dynamics of liver damage and are involved in HBV viral replication, this study focused on the role of genetic variations within genes representing key DNA-repair pathways in HBV-induced liver cirrhosis. The obtained results have demonstrated that SNPs within *XRCC1*, *ERCC2* genes may confer susceptibility to liver cirrhosis in chronic hepatitis B patients.

**Abstract:**

Liver cirrhosis (LC), contributing to more than 1 million of deaths annually, is a major healthcare concern worldwide. Hepatitis B virus (HBV) is a major LC etiological factor, and 15% of patients with chronic HBV infection (CHB) develop LC within 5 years. Recently, novel host genetic determinants were shown to influence HBV lifecycle and CHB course. DNA repair enzymes can affect dynamics of liver damage and are involved in HBV covalently closed circular DNA (cccDNA) formation, an essential step for viral replication. This study aimed to evaluate the possible role of genes representing key DNA-repair pathways in HBV-induced liver damage. MALDI-TOF MS genotyping platform was applied to evaluate variations within *XRCC1*, *XRCC4*, *ERCC2*, *ERCC5*, *RAD52*, *Mre11*, and *NBN* genes. Apart from older age (*p* < 0.001), female sex (*p* = 0.021), portal hypertension (*p* < 0.001), thrombocytopenia (*p* < 0.001), high HBV DNA (*p* = 0.001), and high aspartate aminotransferase (AST) (*p* < 0.001), we found that G allele at rs238406 (*ERCC2*, *p* = 0.025), T allele at rs25487 (*XRCC1*, *p* = 0.012), rs13181 GG genotype (*ERCC2*, *p* = 0.034), and C allele at rs2735383 (*NBN*, *p* = 0.042) were also LC risk factors. The multivariate logistic regression model showed that rs25487 CC (*p* = 0.005) and rs238406 TT (*p* = 0.027) were independently associated with lower risk of LC. This study provides evidence for the impact of functional and potentially functional variations in key DNA-repair genes *XRCC1* and *ERCC2* in HBV-induced liver damage in a Caucasian population.

## 1. Introduction

Liver cirrhosis (LC), the end-stage of different chronic liver diseases, has become one of the major causes of morbidity and mortality with more than 1 million deaths in 2010 according to the Global Burden of Disease (GBD) [1]. As the leading risk factor for the development of hepatocellular carcinoma (HCC), cirrhosis occurs in almost all patients with HCC, being additionally a limiting factor for anticancer therapy of hepatic, as well as non-hepatic malignancies [2]. Although LC is often asymptomatic, there is evidence demonstrating an increase in its prevalence worldwide [3,4]. The growing burden of LC is linked to increasing spread of its risk factors, including hepatitis B virus (HBV) and hepatitis C virus (HCV) infection, as well as non-alcoholic steatohepatitis (NASH) and and alcoholic steatohepatitis (ASH). The Global Hepatitis Report 2017 shows that HBV and HCV infections are responsible for 96% of the global mortality from hepatitis-related end-stage liver disease. In HBV-related cirrhosis, the 5-year cumulative HCC risk is around 15%. 

The natural history of chronic hepatitis B is a complex interplay of viral, host, and environmental factors, and infected individuals can pass through several phases. According to the European Association for the Study of the Liver (EASL), HBeAg-positive chronic HBV infection is the first phase, which is characterized by high HBV DNA, persistent alanine aminotransferase (ALT) levels in upper limits of normal, the presence of serum hepatitis B e antigen (HBeAg), and no or minimal liver inflammation and/or fibrosis [5]. Low rates of spontaneous HBeAg loss are observed in this phase. In HBeAg-positive chronic hepatitis B (phase 2), apart from HBeAg positivity, high viral load and elevated ALT levels, moderate or severe liver inflammation, and/or clinical hepatitis is present [5,6]. Although the outcome of this phase varies, most of patients achieve HBeAg seroconversion and then enter HBeAg-negative chronic HBV infection (phase 3), or HBeAg-negative chronic hepatitis B (phase 4). Following seroconversion from serum antibodies to HBeAg (anti-HBe), HBeAg-negative carriers with undetectable or low levels of viral replication, normal ALT levels, and minimal histological lesions enter phase 3. These patients are at low risk of developing cirrhosis or HCC, but progression to CHB may still occur. Furthermore, a group of patients with moderate to high levels of serum HBV DNA, elevated or fluctuating ALT levels, and active liver necroinflammation and/or fibrosis undergo phase 4. Finally, some patients develop HBsAg-negative phase (phase 5, also known as “occult HBV infection”) with negative serum HBV surface antigen (HBsAg) and positive antibodies to HBV core antigen (anti-HBc), with or without detectable antibodies to HBsAg (anti-HBs). Those who developed cirrhosis before the HBsAg loss are at high risk of HCC as well as HBV reactivation due to immunosuppressive drug treatments [5,6,7].

Chronic liver inflammation affects many cellular pathways including dysregulation of DNA damage response (DDR). DDR plays a critical role in the maintenance of genomic stability and it protects from carcinogenesis by repairing DNA damage caused by multiple factors. Major DNA repair pathways are mismatch repair (MMR), nucleotide excision repair (NER), base excision repair (BER), homologous recombinational repair (HR), and non-homologous end joining (NHEJ). Most of these mechanisms have been previously implicated in HCC, and upregulation of DNA repair genes have been also shown to be associated with cirrhosis [8,9]. Recent experimental data have also linked the cellular DNA repair system with HBV replication cycle. In 2014, Königer et al. identified tyrosyl DNA phosphodiesterase-2 (TDP2) as the first host DNA-repair factor involved in HBV covalently closed circular DNA (cccDNA) biogenesis [10]. However, the additional steps towards cccDNA and the redundancy in DNA repair imply the involvement of numerous others. Moreover, several studies reporting impact of HBV expression on DNA repair support the notion that virus hijacks DDR proteins to successfully complete several steps of its life cycle [11,12,13,14,15]. Furthermore, different DNA tumor viruses have to cope with the DDR through multiple independent mechanisms, and HBV cannot remain an exception [16,17]. Moreover, the HBV interaction with DNA repair was previously suspected because of the association of HCC with chronic hepatitis B (CHB) and the known correlation of cancer with inappropriate DNA damage repair [15]. The interplay between the virus and DDR pathways can modulate to the course of HBV infection. This interaction is mainly determined by the efficiency of DDR machinery, which may be affected by single nucleotide polymorphisms (SNPs) in DNA repair genes [15,18]. These genetic alterations can influence individual DNA repair capacity, increasing the risk of developing liver cirrhosis or cancer, as well as directly affecting the course of HBV infection and HBV-induced pathogenesis. 

Although numerous SNPs in genes encoding DNA repair have been identified, little is known about HBV infection-related DNA repair gene polymorphisms. Therefore, an effective analysis of DNA repair gene polymorphism during viral infection may represent a true advance in the study of HBV-induced liver cirrhosis. In this cross-sectional study, we aimed to determine whether polymorphisms in the key repair enzyme pathways BER (*XRCC1*), NER (*ERCC2*, *ERCC5*), HR (*RAD*52, *Mre11*, *NBN*), and NHEJ (*XRCC4*), which have been previously associated with HBV infection and/or susceptibility to cancer, interfere with HBV-induced liver cirrhosis. 

## 2. Results

### 2.1. Study Group Characteristics

**Figure 1:**
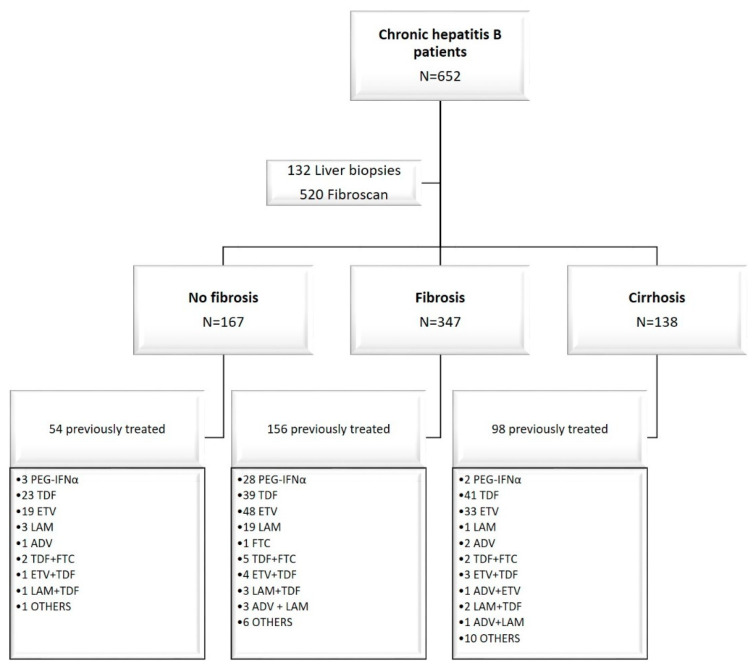
Disposition of enrolled patients. The liver assessment was performed at the time of enrollment in the study, and the treatment history was provided simultaneously. The median of treatment before the liver fibrosis assessment was 3 months. PEG-IFNα, pegylated interferon alfa; TDF, tenofovir; ETV, entecavir; LAM, lamivudine; ADV, adefovir; FTC, emtricitabine; OTHERS: ADV + ETV + LAM + TDF (2), PEG-IFNα + TDF (2), PEG-IFNα + ETV (2), ADV + LAM + TDF (2), vidarabine (1), famciclovir (1), PEG-IFNα + LAM + TDF (2), anti HBsAg monoclonal antibodies (2), anti HBsAg monoclonal antibodies + TDF (2), anti HBsAg monoclonal antibodies + ETV (1).

The study group consisted of 752 participants, including 652 compensated liver disease CHB patients and 100 healthy blood donors. The patients were enrolled in the study at the time of liver assessment. Among CHB patients, 308/652 (47.2%) patients were on antiviral therapy (<6 months prior to study enrollment). These patients received treatment according to Polish or French National Health Service recommendations: 33 (11%) received pegylated interferon alfa (PEG-IFNα), 230 (74.5%) received a nucleoside/nucleotide analogue (NA) monotherapy, and 45 (14.5%) received combined therapies (Figure 1). Table 1 summarizes the distribution of variables evaluated at study inclusion. A total of 138 (21.2%) CHB patients were affected with liver cirrhosis. Patients with liver cirrhosis were significantly older (*p* < 0.001) than patients with and without fibrosis and a control group. Men were predominant in the liver cirrhosis (72%) and fibrosis group (67%). The HBeAg and anti-HBeAg positivity rate in patients with cirrhosis was significantly higher than in participants without cirrhosis (Table 1). 

A total of nine variations within seven genes associated with the DNA repair pathways were successfully genotyped for all study participants (Table S1). The distribution of genotypes followed Hardy–Weinberg equilibrium (HWE) for the healthy volunteers (*p* > 0.05). For CHB patients, the distributions were not consistent with HWE (*p* < 0.05). Genotype distributions for all analyzed SNPs in patients with liver cirrhosis, liver fibrosis, no fibrosis, and healthy group are presented in Tables S2 and S3. Genotypic distribution of 6 analyzed SNPs (*ERCC2*: rs13181, rs238406; *RAD52*: rs7963551; *XRCC1*: rs25487; *ERCC5*: rs2018836; *NBN*: rs2735383) differed significantly between the CHB patients and the healthy control group (Table S2). Moreover, for rs238406 (*ERCC2*), rs25487 (*XRCC1*), and rs2735383 (*NBN*), we also found significant differences in genotypic distribution between the liver cirrhosis and no fibrosis group (Table S3). Evaluation of linkage disequilibrium (LD) pattern with the use of the *r^2^* coefficient between pairs of analyzed SNPs showed that all of them were independent (*r^2^* < 0.5). 

In our study, rs13181 TT (*ERCC2*) and rs2018836 GG (*ERCC5*) genotypes were more common in patients with lower baseline HBV DNA levels (sex-adjusted GG, GT vs. TT: odds ratio (OR) = 1.97, 95% CI 1.35–2.88, *p* = 0.0004; sex-adjusted AA, AG vs. GG: OR = 1.94, 95% CI 1.34–2.81, *p* = 0.00039). *XRCC1* rs25487 TT genotype was associated with lower liver enzyme levels in the study population (ALT: sex-adjusted CC vs. TT: OR = 1.91, 95% CI 1.22–2.99, *p* = 0.0042; AST: sex-adjusted CC vs. TT: OR = 2.26, 95% CI 1.67–4.36, *p* = 0.015). Additionally, carriers of *NBN* rs2735383 CC genotype had significantly lower ALT levels (sex-adjusted GG, GC vs. CC: OR = 1.45, 95% CI 1.05–1.99, *p* = 0.022).

### 2.2. Genetic Polymorphisms and the Susceptibility to CHB-Related Liver Diseases

Three genetic models, additive, dominant, and recessive models, were used to analyze the association between each SNP and liver cirrhosis. Univariate analyses of variables associated with liver cirrhosis are summarized in Table 2, with significant association observed for age, sex, alcohol consumption, treatment experience, hypertension, AST and PLT levels, baseline HBV viral load, rs13181, rs238406, rs25487, and rs2735383. After adjusting for sex and age, logistic regression analysis showed that CHB individuals carrying the rs238406 G allele (*ERCC2*) and rs25487 T allele (*XRCC1*) are at increased risk of developing cirrhosis and liver failure (Table S4). Next, a multiple logistic regression model was used to study the predictors of cirrhosis (Table 3, Table S5). Portal hypertension, thrombocytopenia (platelet count below 150,000), rs25487, and rs238406 were independently associated with liver cirrhosis.

Corresponding analysis was made to investigate associations between evaluated variabilities and the liver fibrosis grade. rs238406 TT (*ERCC2*), rs7963551 TT (*RAD52*), and rs25487 CC (*XRCC1*) were identified as predictors of lower fibrosis stage (S ≤ 2) (Table 4, Table S6). However, rs238406TT and rs25487 CC remained significant predictors in a multiple logistic regression model (Table 5, Table S7). 

## 3. Discussion

In the present study, we investigated the association between the SNPs within DNA repair enzymes associated with HBV infection and/or susceptibility to cancer and HBV-related liver cirrhosis. We showed that SNPs within *XRCC1* and *ERCC2* genes are independently associated with increased risk of cirrhosis. Liver cirrhosis represents the irreversible final fibrosis stage of the liver, being the consequence of wound healing response to chronic liver injury. One of the leading cause of hepatic disease is HBV infection, which was responsible for 66% of viral hepatitis-related deaths in 2015. The unsteady rates of development of liver damage under similar risk factors as well as the variability in the pathogenesis of HBV infection demonstrate that the molecular mechanism of HBV-induced cirrhosis is not fully understood. Recently, a number of functional genetic polymorphisms that likely increase the risk of liver fibrosis progression were described, which, together with other external factors such as alcohol intake, body mass index (BMI), diabetes, or hepatitis could be helpful in the determination of risk profile for the individual patient [19,20,21,22,23,24]. Moreover, host genetic background is undoubtedly involved in the development of HBV-related liver diseases [25]. As HBV exploits the cellular DDR system for RC-DNA to cccDNA conversion, the genes encoding host DNA repair enzymes may be potential candidates to predict the progression of HBV-mediated disease severity [15,18]. 

In this study, we investigated nine genetic polymorphisms in a cohort of CHB patients and in healthy blood donors. We observed significant differences in genotype distribution for six SNPs between CHB patients and healthy individuals. Three genetic variations (*ERCC2*: rs238406, *XRCC1*: rs25487, *NBN*: rs2735383) had diverse frequency in CHB patients with liver cirrhosis and with no fibrosis, highlighting their possible role in HBV-induced liver disease progression. The homozygous TT genotype at *ERCC2* rs238406, CC at *XRCC1* rs25487, and GG at *NBN* rs2735383 were significantly associated with a lower risk of cirrhosis. As compared to the no fibrosis group, the cirrhotic group harbored a higher frequency of *ERCC2* rs238406 G allele, which appeared to be a risk factor for cirrhosis, and lower numbers of the variant *XRCC1* rs25487 C allele, which had a protective effect. Additionally, both alleles together with *RAD52* rs7963551 TT were more common in patients with lower liver fibrosis stage, confirming their role against liver damage progression. 

HBV is a non-cytopathic virus; therefore, liver damage in CHB is associated with host immune response attempting to eliminate the virus [26]. The pathophysiology of HBV-induced fibrosis is related to chronic activation of wound healing mainly as a result of necroinflammation driven by the secondary recruitment of mononuclear cells to the site of infection [27]. Evidence specifically on the role of DNA repair genes in fibrosis progression is scarce. However, the host DNA enzymes and repair machinery is crucial for HBV to replicate. The tyrosyl-DNA phosphodiesterase 2 (TDP2) [10], and more recently reported DNA topoisomerases [28] as well as ataxia telangiectasia mutated pathway and Rad3-related pathway and signaling factor CHK1 (ATR-CHK1) [29], which were shown to take part in the synthesis of HBV cccDNA, may serve as examples. Nonetheless, the detailed mechanisms of HBV DNA turnover, and specifically cccDNA formation, inside the hepatocyte are still to be elucidated, and the possible role of another host factor is postulated [15,18]. Thus, one may speculate that altering the pathways responsible for DNA maintenance might affect viral lifecycle, and, in consequence, relate to the dynamics of generated liver damage. 

The X-ray repair cross complementing 1 protein (XRCC1) plays a crucial role in the coordination of two overlapping repair pathways, SSBR and BER [30]. The *XRCC1* gene, located on chromosome 19q13.2, along with its genetic diversity were widely studied in the past decade. A number of studies reported that *XRCC1* polymorphism is associated with different types of cancer including lung, esophageal, breast, bladder, gastrointestinal, as well as hepatocellular carcinoma [31,32,33,34]. However, results regarding association between rs25487 [C/T] (Arg399Gln) and HCC remain inconclusive. Some studies demonstrated rs25487 as a risk factor of HCC [35,36], whereas others found no such associations [37,38]. In our study, *XRCC1* rs25487 was demonstrated to be a strong prognostic factor for liver cirrhosis and advanced liver fibrosis. To the best of our knowledge, this is the third study where the association between *XRCC1* rs25487 and the risk of HBV-related liver cirrhosis were analyzed. Bose et al. showed that the *XRCC1* genotype alters the risk of HBV-related liver disease [39]. However, this study was conducted on a relatively small group of patients (25 patients with cirrhosis). Another study, performed on Brazilian patients with viral hepatitis, confirmed these findings, but again the study group was relatively small (27 HBV-induced cirrhotic patients) [40]. Even so, both studies present the same hypothesis that *XRCC1* 399 codon has an impact on development of HBV-induced liver cirrhosis. The role of this polymorphism can be explained by the fact that genetic variation at this site contributes to the amino acid change at evolutionarily conserved region, and therefore has an influence on *XRCC1* function [41]. In fact, *XRCC1* rs25487 polymorphism was associated with less efficient DNA repair, which, in consequence, may result in hepatocyte apoptosis [42]. Slyskova et al. demonstrated that individuals with rs25487 TT display a three- to fourfold reduced DNA repair capacity in comparison to CC carriers, which were at lower risk of cirrhosis development in our study [43]. Additionally, the T allele was associated with an increase of chromosomal deletions indicative of defects in BER pathway and with a lower survival time of patients with different types of cancer [44,45,46]. Moreover, in vitro and in vivo studies demonstrated that HBV can induce reactive oxygen species (ROS) production that causes oxidative stress resulting in hepatocyte injury [47,48,49]. As XRCC1 is one of the main players in the repair of ROS-induced lesions [50], it brings us to the conclusion that affecting protein function rs25487 may have an impact on HBV-induced liver fibrosis development. 

The DNA helicase encoded by the excision repair cross-complementing group 2 gene (*ERCC2*), also called xeroderma pigmentosum group D (*XPD*), is involved in basal cellular transcription and NER of DNA lesions [51]. To date, a number of studies have implicated the role of genetic polymorphism within *ERCC2* gene in various cancers, such as gastric, pancreatic, glioma, bladder, and hepatocellular carcinoma [52,53,54,55,56,57]. The most studied polymorphisms rs13181 (Lys751Gln) and rs238406 (Arg156Arg) were demonstrated to alter the function of encoded protein, and, in consequence, influence on DNA repair capacity [58,59]. A recent meta-analysis demonstrated that *ERCC2* rs13181 GG genotype has a positive prognostic value in predicting the overall survival of HCC patients [56]. On the other hand, rs238406 TT genotype was associated with an increased overall cancer risk [60]. In our study, both SNPs were associated with liver cirrhosis development. This effect was stronger for rs238406 G allele, which was identified as a risk factor for both liver cirrhosis and advanced fibrosis. To our knowledge, this is the first study that examines the influence of rs238406 on hepatic cirrhosis. Because rs238406 does not change the amino acid residue (Arg156Arg), it should likely have no effect on the function of ERCC2 enzyme. However, latest studies showed that such a silent mutation may affect gene expression, protein levels, and can also modify the structure and functionality of proteins [61]. Moreover, *ERCC2* expression levels may be used to distinguish between HBV-related HCC tumor and paracancerous tissues [62], which confirms its role in HBV-related liver damage. Moreover, HBV can modulate DNA damage by inhibiting several DNA damage response proteins, including ERCC2, through viral HBx [63]. HBx has shown to directly bind the transcription factor IIH (TFIIH) component xeroderma pigmentosum complementation group D (XPD/ERCC2) [64]. Therefore, any genetic variations within *ERCCA2* may cause structural changes in the interaction site or affect the HBx binding affinity to ERCC2, depressing the DNA repair capability. Very recently, a first connection between genetic variations within ERCC-related genes and cirrhosis was reported [65]. Significant differences in genotype distribiution between control, cirrhosis, and liver cancer groups were demonstrated for *ERCC5* variants. Moreover, it was proposed that two *ERCC5* gene polymorphisms (rs229614 and rs2296148) may be important targets for cirrhosis.

Despite the relatively large sample size, this study has some limitations. First, we did not examine the influence of analyzed SNPs on mRNA levels because of the tissue access constraints. Therefore, the exact mechanisms by which the SNPs regulate the gene transcription activity and/or impact protein activity should be verified. Second, we analyzed only nine SNPs within seven DDR genes, and this may be insufficient to fully predict the DDR contribution to overall risk of HBV-induced liver cirrhosis. Additionally, we lacked some potentially useful characteristics of the patients, such as duration of CHB or phase of infection, which may undoubtedly affect the severity of liver damage. Moreover, as currently noninvasive, elastography-based assessment of liver fibrosis is commonly used in clinical practice, our cohort constituted both patients who underwent liver biopsy and individuals with fibroscan analysis conducted as a noninvasive alternative to study liver fibrosis.

## 4. Materials and Methods

### 4.1. Patients

Adult patients with confirmed CHB infection (HBsAg positive for more than 6 months) were enrolled from the ANRS CO22 HEPATHER cohort (ClinicalTrials.gov registry number: NCT01953458) and Department of Infectious Diseases, Medical University of Gdansk, and the Hepatology Outpatients Clinic Pomeranian Centre for Infectious Diseases and Tuberculosis in Gdansk. Patients were enrolled in the study at the time of liver fibrosis assessment. Only individuals who were treatment-naïve or received only short-term ant-HBV treatment (<6 months) were included in the study. Additionally, a control group containing uninfected subjects seronegative to human immunodeficiency virus (HIV), HBV, and HCV from the Gdansk Regional Centre of Blood Donations and Hemotherapy. 

Patients were diagnosed on the basis of the diagnostic criteria of guidance [66]. All serum samples underwent standard procedure in local clinical center laboratory, including tests for serum HBV surface antigen (HBsAg), HBV surface antibody (HBsAb), HBeAg, HBV e antibody (HBeAb), HBV core antibody (HBcAb), and HBV DNA levels. The patients with co-infection with HIV, HCV, or HDV were excluded from this study. 

Fibrosis scores were assessed when patients were included in the cohort. For 132 patients who had liver biopsy conducted, we assessed degree of fibrosis and inflammation activity according to the Scheuer scoring system (F0–F4) [67]. Non-invasive transient elastography by using FibroScan (Echosens, Paris, France) was used to diagnose liver fibrosis for 520 patients in whom liver biopsy was not performed. Fibrosis and cirrhosis were staged according to the METAVIR scoring system [68]. The liver fibrosis group consisted of the patients who had stage I to stage III fibrosis according to the METAVIR or Scheuer scores. All individuals that had fibrosis stage IV were classified as being in the liver cirrhosis group. 

All procedures followed were in compliance with the Declaration of Helsinki and were approved by the Local Independent Bioethics Committee at the Medical University of Gdansk. All enrolled subjects gave written informed consent for their participation. 

### 4.2. SNP Genotyping

The MagNa Pure LC DNA Isolation System was used for genomic DNA extraction. A sample preparation followed the standard manufacturer protocol for MagNA Pure Compact Nucleic Acid Isolation Kit I (Roche, Mannheim, Germany). Gene analysis of *XRCC1* (rs25487), *ERCC2* (rs238406, rs13181), *ERCC5* (rs2018836), *RAD*52 (rs7963551), *Mre11* (rs496797, rs535801)*, NBN* (rs2735383), and *XRCC4* (rs1805377) was carried out by mass spectrometry analysis. The Mass Assay Designer software package (v.4.0) was used to design 9 specific PCR primer pairs and 9 extension primer sequences (Table S1). The manufacturer’s standard protocol was followed for iPLEX Pro chemistry (Agena Bioscience, San Diego, CA, USA). In brief, genomic DNA was amplified resulting in 10 different products containing SNPs of interest, followed by a shrimp alkaline phosphatase treatment to remove the excess of nucleotides. Next, the single-base extension reaction was performed with mass-modified terminator nucleotides, and after desalting, the obtained products were dispensed onto a 96 SpectroCHIP array using an RS1000 Nanodispenser. SNPs variations were distinguished with MALDI-TOF mass spectrometry on the basis of different molecular weights and analyzed by MassARRAY Typer Analyzer 4.0 software. 

### 4.3. Statistical Analysis

Statistical analyses were performed using STATISTICA software version 13.3 (StatSoft, Tulsa, OK, USA). The linkage disequilibrium analysis and deviations from Hardy–Weinberg equilibrium of analyzed SNPs were conducted by the MIDAS software. The relation between categorical vs. categorical variables were evaluated by chi-squared or Fisher’s exact test, as appropriate. Logistic regression analysis was used to evaluate the contribution of genetic and nongenetic factors under the dominant, recessive, and additive models. A backward stepwise regression approach was applied when building multivariate models. All of the *p*-values presented were two-sided and only *p* < 0.05 was considered significant. A Benjamini–Hochberg procedure was applied to account for multiple testing. Statistical power was calculated post hoc using the G*Power 3.1.9.4.

## 5. Conclusions

In conclusion, this study shows that functional and potentially functional SNPs within *XRCC1* and *ERCC2* genes may confer susceptibility to HBV-associated liver cirrhosis. Moreover, *ERCC2* rs238406, acting as a biomarker of high liver fibrosis stage, suggests an important role of the functional *ERCC2* SNPs in the etiology of HBV-induced liver damage in a Caucasian population. Considering limitations of this study, future studies should explore more genes, more SNPs, and interaction with environmental/behavioral factors such as the alcohol use.

## Figures and Tables

**Table 1:** Characteristics of chronic hepatitis B patients with liver cirrhosis, hepatic fibrosis, and healthy controls.

Variable	Chronic Hepatitis B			*p*-Value ^d^	Healthy Controls (*n* = 100)	*p*-Value ^e^
	No Fibrosis ^a^ (*n* = 167)	Liver Fibrosis ^b^ (*n* = 347)	Liver Cirrhosis ^c^ (*n* = 138)			
Age, years	46 ± 1	50 ± 1	60 ± 0.9	<0.0001	27.3 ± 0.9	<0.0001
Sex, % females	49%	33%	28%	0.00148	22%	0.0031
Ethnicity, % Caucasian	100%	100%	100%	1	100%	1
Alcohol consumption, %	54%	55%	34%	0.00015	-	-
Previous treatment, %	32%	50%	71%	<0.0001	-	-
BMI, kg/m^2^	25.37 ± 0.39	25.67 ± 0.29	26.20 ± 0.41	0.1752	-	-
Diabetes, %	5%	11%	12%	0.0175	-	-
HT, %	15%	24%	99%	<0.0001	-	-
ALT, IU/L	32.73 ± 1.64	37.43 ± 1.58	40.43 ± 2.71	0.0070	-	-
AST, IU/L	26.37 ± 1.17	29.68 ± 1.34	38.4 ± 2.2	<0.0001	-	-
ALB, IU/L	41 ± 1.06	43.6 ± 0.25	40.8 ± 0.48	0.4838	-	-
PLT, 10^9^/L	229 ± 4.58	208 ± 3.85	158 ± 6.06	0.0002	-	-
HBV DNA, IU/mL	8447 ± 3 828	15,015 ± 3 787	20,932 ± 269	<0.0001	-	-
HBeAg, % positive	6%	13%	18%	0.0014	-	-
Anti-HBe, % positive	92%	82%	66%	<0.0001	-	-
Liver inflammation grade ^†,^*	1.5 (0–2)	1.5 (1–3)	1.5 (1.25–1.75)	0.3017	-	-
Liver fibrosis stage ^†^	0 (0–0)	1 (0.5–3.5)	4 (4–4)	<0.0001	-	-

Unless stated otherwise, data are shown as the mean ± standard error of the mean; **^†^** median value (interquartile range); * liver biopsy samples only (*n* = 132); **^a^** no scarring (stage 0); **^b^** fibrosis stages (I–III); **^c^** fibrosis stage IV; ^d^ liver cirrhosis vs. no fibrosis, ^e^ all CHB vs. healthy control group; *p*-values less than 0.05 are shown in bold. BMI, body mass index; HT, portal hypertension; ALT, alanine aminotransferase; AST, aspartate aminotransferase; ALB, albumin; PLT, platelet count; CHB, chronic hepatitis B.

**Table 2:** Results of univariate analyses for liver cirrhosis risk in chronic hepatitis B patients.

Variable	*n* (%)	OR	95% CI	*p-*Value
**Age** >50 years ≤50 years	346 (53%) 306 (47%)	4.19	2.71–6.48	**<0.001**
**Sex** Female Male	242 (37%) 410 (63%)	1.59	1.07–2.36	**0.021**
**Alcohol consumption** YES NO	332 (51%) 320 (49%)	2.37	1.58–3.55	**<0.001**
**Previous treatment** YES NO	308 (47%) 344 (53%)	2.64	1.73–4.02	**<0.001**
**Portal hypertension** YES NO	254 (39%) 398 (61%)	515	71–3756	**<0.001**
**AST** >50 IU/L ≤50 IU/L	118(18%) 534 (82%)	3.10	1.76–58.44	**<0.001**
**ALT** >42 IU/L ≤42 IU/L	98 (15%) 554 (85%)	1.22	0.80–1.84	0.34
**PLT** >150 IU/L ≤150 IU/L	502 (77%) 150 (23%)	4.96	3.18–7.73	**<0.001**
**HBV DNA** >6000 kIU/mL ≤6000 kIU/ml	529 (81%) 123 (19%)	3.75	1.70–8.26	**0.001**
**AST/ALT** >1 ≤1	254 (39%) 398 (61%)	1.73	1.18–2.53	**0.004**
***XRCC1* rs25487**				
CC vs. TT	294 (74%) vs. 104 (26%)	1.58	0.95–2.60	0.069
CC vs. CT, TT	294 (45%) vs. 358 (55%)	0.61	0.42–0.89	**0.012**
TT vs. CT, CC	104 (16%) vs. 548 (84%)	1.03	0.63–1.70	0.89
***ERCC2* rs238406**				
GG vs. TT	235 (60.5%) vs. 154 (39.5%)	1.58	0.91–2.72	0.099
GG vs. GT, TT	235 (36%) vs. 417 (64%)	1.14	0.75–1.73	0.54
TT vs. GT, GG	154 (23.5%) vs. 498 (76.5%)	0.53	0.30–0.92	**0.025**
***ERCC2* rs13181**				
TT vs. GG	303 (72%) vs. 117 (28%)	4.27	1.11–16.42	**0.034**
GG vs. GT, TT	117 (18%) vs. 535 (82%)	1.35	0.86–2.14	0.19
TT vs. GT, GG	303 (46.5%) vs. 349 (53.5%)	0.91	0.63–1.33	0.64
***ERCC5* rs2018836**				
GG vs. AA	333 (79%) vs. 88 (21%)	1.28	0.77–2.12	0.33
GG vs. GA, AA	333 (51%) vs. 319 (49%)	1.41	0.86–2.30	0.17
AA vs. GA, GG	88 (13.5%) vs. 564 (86.5%)	0.92	0.61–1.32	0.65
***RAD 52* rs7963551**				
TT vs. GG	492 (87%) vs. 72 (13%)	1.04	0.80–1.25	0.97
TT vs. TG, GG	492 (75.5%) vs. 160 (24.5%)	0.97	0.61–1.54	0.91
GG vs. TG, TT	72 (11%) vs. 580 (89%)	0.96	0.51–1.81	0.90
***MRE 11* rs496797**				
TT vs. CC	163 (44%) vs. 209 (56%)	0.67	0.42–1.09	0.11
TT vs. TC, CC	163 (25%) vs. 489 (75%)	1.17	0.78–1.76	0.44
CC vs. TC, TT	209 (32%) vs. 443 (68%)	0.68	0.45–1.003	0.067
***MRE 11* rs535801**				
CC vs. TT	316 (77%) vs. 95 (23%)	1.10	0.67–1.82	0.70
CC vs. CT, TT	316 (48.5%) vs. 336 (51.5%)	1.04	0.72–1.50	0.84
TT vs. CT, CC	95 (14.5%) vs. 557 (85.5%)	1.30	0.80–2.10	0.29
***NBN* rs2735383**				
CC vs. GG	336 (77%) vs. 101 (23%)	0.59	0.35–1.01	0.056
CC vs. CG, GG	336 (51.5%) vs. 316 (48.5%)	1.29	0.88–1.89	0.18
GG vs. CG, CC	101 (15.5%) vs. 551 (84.5%)	0.53	0.29–0.98	**0.042**
***XRCC4* rs1805377**				
GG vs. AA	522 (97.5%) vs. 13 (2.5%)	1.51	0.71–3.21	0.28
GG vs. GA, AA	522 (80%) vs. 130 (20%)	0.85	0.54–1.32	0.46
AA vs. GA, GG	13 (2%) vs. 639 (98%)	2.15	7.44–6.22	0.16

*p*-values less than 0.05 are marked in bold. CI, confidence interval; OR, odds ratio.

**Table 3:** Final multiple logistic regression model for liver cirrhosis risk in chronic hepatitis B patients.

Variable	*n* (%)	OR	95% CI	*p-*Value
**Portal hypertension** YES NO	254 (39%) 398 (61%)	446	58–3425	**<0.001**
**Previous treatment** YES NO	308 (47%) 344 (53%)	3.88	1.56–9.67	**0.003**
**PLT** >150 IU/L ≤150 IU/L	502 (77%) 150 (23%)	4.76	1.97–11.67	**<0.001**
***XRCC1* rs25487**				
CC vs. CT, TT	294 (45%) vs. 358 (55%)	0.27	0.13–0.57	**0.005**
***ERCC2* rs238406**				
TT vs. GT, GG	154 (23.5) vs. 498 (76.5)	0.32	0.12–0.88	**0.027**

*p*-values less than 0.05 are marked in bold.

**Table 4:** Results of univariate analyses for the risk of advanced liver fibrosis ^†^ in chronic hepatitis B patients.

Variable	*n* (%)	OR	95% Cl	*p-*Value
**Age** >50 years ≤50 years	346 (53%) 306 (47%)	2.98	2.15–4.12	**<0.001**
**Sex** Female Male	242 (37%) 410 (63%)	0.39	0.28–0.55	**<0.001**
**Alcohol consumption** YES NO	332 (51%) 320 (49%)	1.70	1.21–2.40	**0.002**
**Previous treatment** YES NO	308 (47%) 344 (53%)	3.19	2.23–4.57	**<0.001**
**Portal hypertension** YES NO	254 (39%) 398 (61%)	7.32	4.98–10.76	**<0.001**
**AST** >50 IU/L ≤50 IU/L	118 (18%) 534 (82%)	3.76	2.07–6.84	**<0.001**
**ALT** >42 IU/L ≤42 IU/L	98 (15%) 554 (85%)	0.62	0.44–0.87	**0.006**
**PLT** >150 IU/L ≤150 IU/L	502 (77%) 150 (23%)	6.64	4.11–10.74	**<0.001**
**HBV DNA** >6000 kIU/mL ≤6000 kIU/mL	529 (81%) 123 (19%)	1.76	1.13–2.73	**0.011**
**AST/ALT** >1 ≤1	254 (39%) 398 (61%)	1.44	1.02–2.04	**0.036**
**XRCC1 rs25487**				
CC vs. TT	294 (45) vs. 104 (16)	1.26	0.82–1.94	0.29
CC vs. CT, TT	294 (45%) vs. 358 (55%)	0.69	0.51–0.95	**0.024**
TT vs. CT, CC	104 (16%) vs. 548 (84%)	0.78	0.49–1.22	0.27
**ERCC2 rs238406**				
GG vs. TT	235 (36) vs. 154 (23.5)	0.92	0.59–1.42	0.70
GG vs. GT, TT	235 (36) vs. 417 (64)	1.04	0.76–1.42	0.79
TT vs. GT, GG	154 (23.5) vs. 498 (76.5)	0.91	0.97–1.44	0.68
**ERCC2 rs13181**				
TT vs. GG	303 (46.5) vs. 117 (17)	2.85	0.66–12.39	0.16
GG vs. GT, TT	117 (18) vs. 535 (82)	1.17	0.78–1.76	0.43
TT vs. GT, GG	303 (46.5) vs. 349 (53.5)	0.90	0.66–1.23	0.53
**ERCC5 rs2018836**				
GG vs. AA	333 (51) vs. 88 (13.5)	0.73	0.47–1.14	0.17
GG vs. GA, AA	333 (51) vs. 319 (49)	1.04	0.73–1.49	0.80
AA vs. GA, GG	88 (13.5) vs. 564 (86.5)	0.64	0.42–0.96	**0.031**
**RAD 52 rs7963551**				
TT vs. GG	492 (75.5) vs. 72 (11)	1.67	1.04–2.68	**0.032**
TT vs. TG, GG	492 (75.5) vs. 160 (24.5)	0.66	0.46–0.97	**0.034**
GG vs. TG, TT	72 (11) vs. 580 (89)	1.54	0.94–2.54	0.08
**MRE 11 rs496797**				
TT vs. CC	163 (25) vs. 209 (32)	0.94	0.63–1.42	0.79
TT vs. TC, CC	163 (25) vs. 489 (75)	0.88	0.62–1.26	0.49
CC vs. TC, TT	209 (32) vs. 443 (68)	0.83	0.59–1.16	0.28
**MRE 11 rs535801**				
CC vs. TT	316 (48.5) vs. 95 (14.5)	0.86	0.56–1.30	0.47
CC vs. CT, TT	316 (48.5) vs. 336 (51.5)	1.15	0.84–1.57	0.37
TT vs. CT, CC	95 (14.5) vs. 557 (85.5)	0.95	0.62–1.46	0.83
**NBN rs2735383**				
CC vs. GG	336 (51.5) vs. 101 (15.5)	1.03	0.68–1.57	0.88
CC vs. CG, GG	336 (51.5) vs. 316 (48.5)	0.88	0.64–1.21	0.45
GG vs. CG, CC	101 (15.5) vs. 551 (84.5)	0.55	0.55–1.31	0.46
**XRCC4 rs1805377**				
GG vs. AA	522 (80) vs. 13 (2)	1.23	0.63–2.39	0.54
GG vs. GA, AA	522 (80) vs. 130 (20)	0.84	0.57–1.24	0.39
AA vs. GA, GG	13 (2) vs. 639 (98)	0.81	0.27–2.41	0.71

CI, confidence interval; MAF, minor allele frequency. *p*-values less than 0.05 are marked in bold. ^†^ S > 2.

**Table 5:** Results of multivariate analyses for advanced ^†^ liver fibrosis.

Variable	*n* (%)	OR	95% Cl	*p-*Value
**Sex** Female Male	242 (37%) 410 (63%)	0.48	0.28–0.84	**0.009**
**Previous treatment** YES NO	308 (47%) 344 (53%)	2.30	1.40–3.78	**<0.001**
**Portal hypertension** YES NO	254 (39%) 398 (61%)	6.60	4.00–10.89	**<0.001**
**AST** >50 IU/L ≤50 IU/L	118 (18%) 534 (82%)	2.44	1.06–5.62	**0.034**
**PLT** >150 IU/L ≤150 IU/L	502 (77%) 150 (23%)	3.84	2.02–7.26	**<0.001**
***XRCC1* rs25487**				
CC vs. CT, TT	294 (45%) vs. 358 (55%)	0.51	0.31–0.85	**0.009**
***ERCC2* rs238406**				
TT vs. GT, GG	154 (23.5) vs. 498 (76.5)	0.47	0.27–0.81	**0.006**

CI, confidence interval; OR, odds ratio; *p*-values less than 0.05 are marked in bold. ^†^ S > 2. *p*-values less than 0.05 are marked in bold

## Supplementary Materials

The following are available online at https://www.mdpi.com/2072-6694/12/11/3295/s1. Table S1: Primers used for MALDI-TOF MassARRAY single-nucleotide polymorphism genotyping. Table S2: Genotypic distribution of analyzed SNPs among chronic hepatitis B patients and a healthy control group. Table S3: Genotypic distribution of analyzed SNPs among chronic hepatitis B patients with liver cirrhosis, hepatic fibrosis, and a no fibrosis group. Table S4: Multiple logistic regression model for liver cirrhosis risk in chronic hepatitis B patients. Table S5: Mann-Whitney *U*-test analysis levels of selected variabilities in the plasma of patients with and without liver cirrhosis. Table S6: Mann-Whitney *U*-test analysis levels of selected variabilities in the plasma of patients with and without advanced liver fibrosis. Table S7: Multiple logistic regression model for liver cirrhosis risk in chronic hepatitis B patients.
